# Effects of Particulate Matter and Its Chemical Constituents on Elderly Hospital Admissions Due to Circulatory and Respiratory Diseases

**DOI:** 10.3390/ijerph13100947

**Published:** 2016-09-23

**Authors:** Tatiane Morais Ferreira, Maria Cristina Forti, Clarice Umbelino de Freitas, Felipe Parra Nascimento, Washington Leite Junger, Nelson Gouveia

**Affiliations:** 1 Pos-Graduate Program in Environmental Science, University of São Paulo, São Paulo SP 05508-010, Brazil; tmoraisf@gmail.com; 2 Center of Earth System Science, National Institute for Space Research, São José dos Campos SP 12227-010, Brazil; cristina.forti@inpe.br; 3 Department of Preventive Medicine, School of Medicine, University of São Paulo, São Paulo SP 01246-903, Brazil; umbelino.freitas@gmail.com (C.U.d.F.); fepnascimento@gmail.com (F.P.N.); 4 Department of Epidemiology, Institute of Social Medicine, State University of Rio de Janeiro, Rio de Janeiro RJ 20550-900, Brazil; wjunger@ims.uerj.br

**Keywords:** air pollution, chemical constituents, hospital admission, particulate matter, time series

## Abstract

Various fractions of particulate matter have been associated with increased mortality and morbidity. The purpose of our study is to analyze the associations between concentrations of PM_2.5_, PM_2.5–10_, PM_10_ and their chemical constituents (soluble ions) with hospital admissions due to circulatory and respiratory diseases among the elderly in a medium-sized city in Brazil. A time series study was conducted using Poisson regression with generalized additive models adjusted for confounders. Statistically significant associations were identified between PM_10_ and PM_2.5–10_ and respiratory diseases. Risks of hospitalization increased by 23.5% (95% CI: 13.5; 34.3) and 12.8% (95% CI: 6.0; 20.0) per 10 μg/m^3^ of PM_2.5-10_ and PM_10_, respectively. PM_2.5_ exhibited a significant association with circulatory system diseases, with the risk of hospitalization increasing by 19.6% (95% CI: 6.4; 34.6) per 10 μg/m^3^. Regarding the chemical species; SO_4_^2−^, NO_3_^−^, NH_4_^+^ and K^+^ exhibited specific patterns of risk, relative to the investigated outcomes. Overall, SO_4_^2−^ in PM_2.5–10_ and K^+^ in PM_2.5_ were associated with increased risk of hospital admissions due to both types of diseases. The results agree with evidence indicating that the risks for different health outcomes vary in relation to the fractions and chemical composition of PM_10_. Thus, PM_10_ speciation studies may contribute to the establishment of more selective pollution control policies.

## 1. Introduction

The association between inhaled particulate matter (PM_10_), i.e., particles with aerodynamic diameter ≤10 µm, and harmful health effects is a relevant public health problem that has been widely documented in epidemiological studies carried out in various parts of the world. The effects associated with exposure to PM_10_ range from hospital admissions to deaths from respiratory and cardiac diseases, particularly among the most vulnerable population groups, such as children and the elderly [1,2,3].

PM_10_ is an indicator of air pollution with high physical-chemical complexity and exhibits peculiar characteristics as a function of the sources of emission. Essentially, particulate matter is classified as coarse (PM_2.5–10_) or fine (PM_2.5_) particles, which allows for its deposition in several parts of the respiratory system; eventually reaching the circulatory system [4]. Harmful effects are associated with the various PM_10_ fractions [1,5,6]. However, it is believed that some of its chemical species are able to significantly modify the association of PM_10_ with hospital admissions [7,8,9].

Among the chemical constituents that appear to pose greater risks to health are those derived from the burning of biomass and fossil fuels [3]. Some studies suggest that the risk PM_10_ poses to health is due to its carbonaceous fraction and metals [5,10,11,12]. However, some relevant institutions recently reviewed articles on toxicology and concluded that, while black carbon might not be the main toxic component, it might behave as a universal carrier of chemical constituents to sensitive targets in the human body. In addition, inorganic constituents such as SO_4_^2−^ and NO_3_^−^ might possibly have interactive biological effects with other constituents, thus influencing the bioavailability of definite components [13]. Son and colleagues reported an association between exposure to soluble inorganic constituents and mortality by respiratory and cardiovascular causes in Seoul, South Korea, but they did not find such an association relative to the carbonaceous fraction, although it represented 32% of the PM_2.5_ mass [8].

The chemical composition of PM_10_ varies over time and by location, as a function of the sources of emission, which does not allow the results obtained at any given place and time to be generalized [10]. Thus far, most of the studies examining how the chemical constituents of particles relate to health were conducted in North America and Europe and this reinforces the need to assess the impact of pollution on health as a function of the chemical constituents of each PM_10_ fraction in different locations.

The studies conducted in Latin America up to the present time have assessed the risk associated with particulate matter total mass only and mainly in large cities [14]. Therefore, the aim of the present study was to investigate the associations between the concentrations of PM_2.5_, PM_2.5–10_, PM_10_, and their soluble inorganic chemical constituents, and hospital admissions of elderly people due to respiratory and circulatory diseases in a midsized city in Brazil.

## 2. Materials and Methods

### 2.1. Study Setting

São José dos Campos (SJC) is located at 23°10’47”S and 45°53’14”W and is 91 km away from the capital of the state of São Paulo. This area is a seat for automobile, chemical-pharmaceutical, petrochemical and aerospace industries and contains several highways with high traffic density.

The air quality is monitored by the Environmental Company of the State of São Paulo (Companhia Ambiental do Estado de São Paulo—CETESB) based on an automatic station for measuring PM_10_, SO_2_, O_3_, relative humidity, temperature, wind speed and direction and a manual station to monitor smoke.

The municipal healthcare network includes three hospitals, five emergency care units, and 40 basic and 16 specialized healthcare units, in addition to contracted and associated private hospitals. The Unified Health System (Sistema Único de Saúde—SUS) provides care to approximately 400,000 people (60% of the population) [15].

### 2.2. Data on Hospital Admissions

Data on hospital admissions were available from computerised files of the Health Services Information Database (DATASUS) of the Brazilian Unified Health System (SUS) [16]. Such data are processed and made available by DATASUS and can be used for health situation analysis, decision-making and the elaboration of health programs. These data consisted of information on date of birth, age and sex, date of admission and discharge, and the main diagnosis for the admission (coded according to the International Classification of Diseases, 10th revision: ICD-10) besides other information. Daily hospital admissions due to respiratory (J00–J99) and circulatory diseases (I00–I99) in elderly people (≥60 years old) admitted from 5 March 2010 to 17 February 2011 were included for analysis.

### 2.3. Environmental Data

The data relative to the mass concentrations of PM_2.5_, PM_2.5–10_, and the soluble fractions of larger cations (Na^+^, NH_4_^+^, K^+^, Ca^2+^ and Mg^2+^) and larger anions (Cl^−^, NO_3_^−^ and SO_4_^2−^) were provided by the Laboratory of Environmental Research on Aerosols, Aqueous Solutions and Technologies (Laboratório de Pesquisa Ambiental em Aerossóis, Soluções Aquosas e Tecnologias—LAQUATEC) and the National Institute for Space Research (Instituto Nacional de Pesquisas Espaciais—INPE).

Samples of particulate matter were obtained by means of a Gent Stacked Filter*-*like sampler, which separates the PM_2.5_ and PM_2.5–10_ fractions of PM_10_ [17]. The PM_10_ concentration corresponds to the sum of the concentration of both fractions (PM_2.5_ and PM_2.5–10_). The procedures, relative to the sampling system, handling, and determination of PM_10_ mass, are described in full detail in Ferreira and colleagues [18]. To obtain the soluble fractions, the sampled filters were solubilized and analyzed by ion chromatography (Metrohm-850 Professional IC, Metrohm, Herisau, Switzerland). The analytical procedures to obtain soluble ions, storage, calculation of the limits of detection and quantification and validation of the method used in the chemical analysis of the samples are described in the INPE Protocols [19,20,21].

Data on PM_10_ were also obtained from the CETESB monitoring station. The automatic station, at which the measurements are performed using the beta radiation method, is linked to a central computer via a telemetry system that continuously records atmospheric concentrations. Based on that station, meteorological data, relative to the temperature and humidity, were also used. The station is located approximately 2.6 km away from INPE.

### 2.4. Data Analysis

Time series of daily counts were used to assess the effects of exposure to air pollution, on the same day and a few days before, on health. Measurements of the pollutants were missing for days on which particulate matter was not collected. Therefore, missing data imputation was carried out by means of a procedure available in the *mtsdi* library R software version 3.0.2 (R Development Core Team, 2013, R Foundation, Vienna, Austria). The method for imputation is based on the EM (expectation-maximization) algorithm, which considers the dependence among variables and the temporal dependence of each variable [22]. Unidentified chemical species in the samples were imputed with half the limit of quantification of the method for each one of the chemical constituents.

The time series analysis was performed by means of Poisson regression using generalized additive models (GAM), which allow for non-linear terms. In the Poisson regression, the response is the natural logarithm of the expected number of events on any given day [23]. Thus, the following model was assumed:

(1) $$\ln(E[Y|X])=\beta_{0}+\sum_{j}\beta_{j}X_{j}+\sum_{i}S_{i}(X_{i}),$$

where E[Y|X] is the expected number of elderly hospital admissions conditional on the covariates on a given day, $X_{j}$ are the linear terms, $\beta_{j}$ the regression coefficients of each linear term, and $S_{i}$ are smooth functions of the covariates $X_{i}$.

The analysis was based on the identification of a baseline model. Indicator variables were used to control the effects of weekdays and bank holidays, and natural cubic spline smoothing [23] was used for the confounding variables (temperature and relative humidity) and time series structures (trend and seasonality). Three and two degrees of freedom (df) were used to model the minimum temperature and minimum humidity, respectively. For the time series structures, four and three df were used for respiratory and circulatory diseases models. The number of days between the measurement of temperature and humidity and the occurrence of the outcome, as well as the smoothing parameter for the natural cubic splines, were chosen in such a way that the fitted model shows the lowest value for the Akaike’s information criterion (AIC).

Once the baseline model was established, the daily concentrations of the exposure variables were added to the model, assuming a linear relationship with the response variable. The relative risk (RR) estimates were obtained based on a polynomial distributed lag model (PDLM) for up to 5 days, which accounts for the joint distribution of adjacent lags in this time-frame [24]. For the constraining polynomial base, we used 2 df.

The results represent the percentage increase in risk of hospital admission for a 10 μg/m^3^ increase for PM_10_ and its fractions, and 10 ng/m^3^ for the chemicals’ constituents, whereby the percentage of RR for a 10-unit increase was estimated as

(2) $$\%RR=(e^{10\beta }-1)\times 100,$$

where β is the regression coefficient of the exposure variable. The significance level was set to 5%.

Data analysis was performed using R software version 3.0.2 with the Air and Health (ARES) library, which was developed to conduct time series analysis of the effects of air pollution on health [25].

## 3. Results

The data obtained from INPE relative to PM_10_ fractions and chemical constituents corresponded to 80% and 75%, respectively, of the study period (350 days). In turn, the data on PM_10_ provided by the CETESB station corresponded to the full (100%) study period.

**Figure 1:**
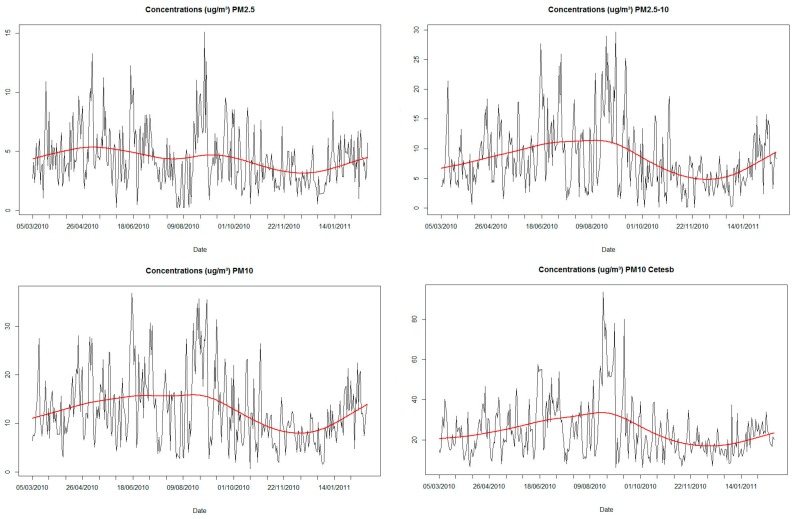
The 24-hour concentrations of pollutants, in μg/m^3^, as measured by the National Institute for Space Research (Instituto Nacional de Pesquisas Espaciais—INPE) and CETESB monitoring systems. Line, smoothing spline with 6 degrees of freedom.

The results of descriptive analysis in terms of the pollutants, meteorological variables and hospital admission are described in Table 1. The 24-hour and annual average concentrations of PM_10_ and PM_2.5_ did not exceed the standards established by the World Health Organization, WHO (PM_10_ 24-hour: 50 μg/m^3^; PM_10_ annual: 20 μg/m^3^; PM_2.5_ 24-hour: 25 μg/m^3^; PM_2.5_ annual: 10 μg/m^3^) [26]. Due to a difference inherent to each of the collection systems used, as well as the collection height, the values of data collected by the CETESB station were approximately twice as high as those that were in the INPE data collection system; nevertheless, the distribution of the PM_10_ concentration levels was similar in both (Figure 1), whose correlation was 0.85. The maximal 24-hour and annual average concentrations of PM_10_ were 93.6 μg/m^3^ and 24.5 μg/m^3^, respectively, levels that were above the limits established by the WHO [26]. Although the smooth curve did not detect any long-term trend, greater concentration levels of the assessed fractions occurred in the dry season (April to August) and decreased in the rainy season, except for PM_2.5_, for which the concentration also decreased in the rainy season, although the variation of this fraction over time was less remarkable (Figure 1).

Soluble ions represented 17% and 11% of the PM_2.5_ and PM_2.5–10_ masses, respectively, along the study period. The order of contribution of PM_2.5_ constituents was as follows: SO_4_^2−^ > NH_4_^+^ > NO_3_^−^ > Ca^2+^ > Na^+^ > Cl^−^ > K^+^ > Mg^2+^. Relative to PM_2.5-10_, the corresponding order was as follows: NO_3_^−^ > Ca^2+^ > SO_4_^2−^ > Cl^−^ >Na^+^ > NH_4_^+^ > K^+^ > Mg^2+^ (Table 2).

**Figure 2:**
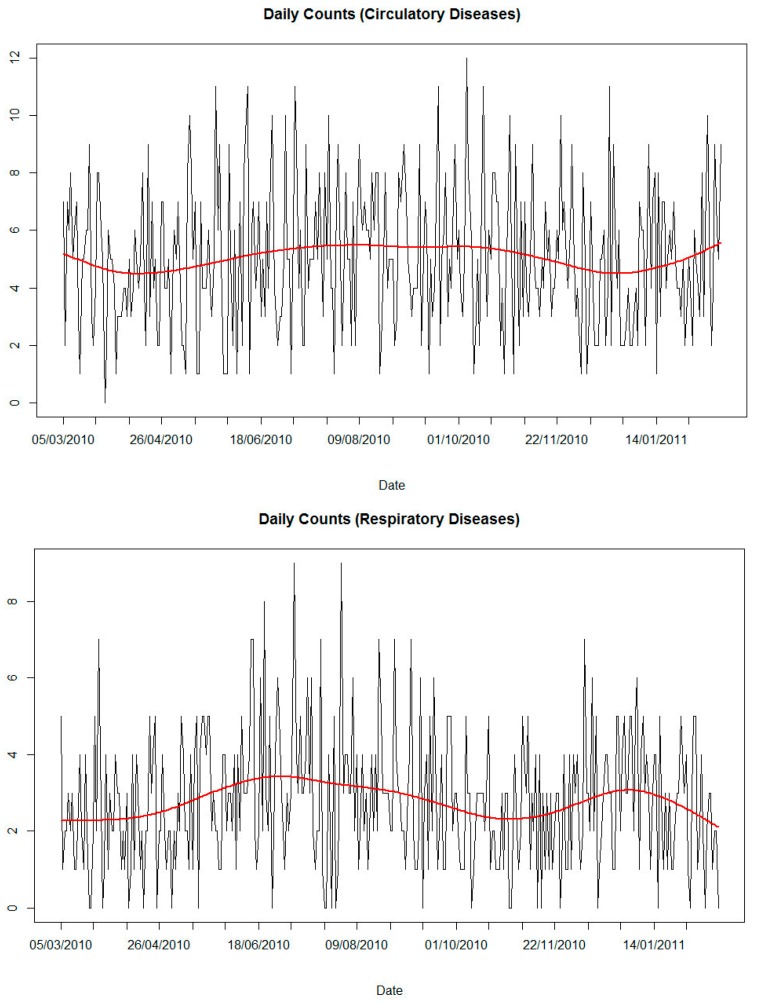
Number of hospital admissions of elderly people (≥60 years old) due to circulatory and respiratory diseases per day from 5 March 2010 to 17 February 2011. Line, smoothing spline with 3 degrees of freedom relative to the circulatory diseases and 4 degrees of freedom relative to the respiratory diseases.

The number of hospital admissions due to circulatory diseases (n = 1765) was higher than the number of admissions due to respiratory diseases (n = 972). The average number of hospital admissions per day was five for circulatory and three for respiratory diseases (Table 1). The distribution of hospital admissions throughout the study period is depicted in Figure 2. No striking long-term trend or seasonality pattern was detected; however, the number of admissions due to respiratory diseases was higher in the dry season, while the distribution of circulatory system diseases seemed to be less variable over time.

The estimates of the effects of each pollutant on hospital admissions are described in Table 3. Statistically significant associations were identified between PM_10_ and PM_2.5–10_ and respiratory diseases and between PM_2.5_ and circulatory diseases. The estimates based on the CETESB station data were similar to those based on the INPE data, whose risk for hospital admission was detected only for respiratory diseases.

Only the estimates of the chemical constituents detected by chemical analysis in at least 75% of the study period were considered to be reliable. In regard to PM_2.5–10_ components, positive, statistically significant associations were identified between SO_4_^2−^ and respiratory diseases and between SO_4_^2−^, NO_3_^−^ and K^+^ and circulatory diseases. Relative to PM_2.5_, only SO_4_^2−^, NH_4_^+^ and K^+^ met the conditions established for valid estimates. SO_4_^2−^ and NH_4_^+^ were associated with a risk of hospital admission due to circulatory diseases, and K^+^ was associated with both causes of hospital admission (Table 3).

## 4. Discussion

To our knowledge, this is the first study of the relationship between particulate matter fractions and chemical composition and health effects in a Latin America city. Results indicate an increased risk of hospitalization of elderly residents for respiratory diseases with increasing PM_10_ and PM_2.5–10_ concentrations and for circulatory diseases with increasing PM_2.5_ concentration. Statistically significant positive associations were also observed between particulate constituents (SO_4_^2−^, NH_4_^+^, NO_3_^−^ and K^+^) in each of the fractions and both examined outcomes in this medium-sized city in Brazil.

The effects for all fractions of PM_10_ were of larger magnitude than those usually reported in the literature for the USA and Europe [27,28,29]. For example, a 10 μg/m^3^ increase in PM_10_ was associated with a 12.8% (95% CI: 6.0; 20.0) and 8.9% (95% CI: 5.2; 12.8) increase in hospital admissions for respiratory diseases based on the data provided by INPE and CETESB, respectively. Although studies conducted in other cities in Brazil have reported smaller effect estimates [30], our results are consistent with a previous study on the hospitalization of the elderly carried out in the same city [31].

Although most research and regulatory agencies have directed their efforts and attention to the fine fraction of PM_10_, it is recognized that a reduction in the coarse fraction can also bring important health benefits [6,32]. However, examination of the acute effects of PM_2.5–10_ on hospital admissions is still relatively rare compared to PM_2.5_ and results are somehow inconsistent [33]. Recently, a study involving 35 cities in California, USA, identified a positive association between PM_2.5–10_ and emergency visits for respiratory disease even after adjustment for PM_2.5_ [32]. It should be noted that in our study, coarse particles were only associated with respiratory admissions and this fraction represented 66% of the total PM mass in the studied city. This suggests the importance of monitoring and assessing different fractions of atmospheric PM_10_ in this context.

Regarding the effects of the PM_2.5–10_ chemical constituents, we detected statistically significant associations between SO_4_^2−^ and both circulatory and respiratory hospitalizations and between NO_3_^−^ and K^+^ and circulatory hospitalizations only. We could not identify any study of the health effects of soluble ions of the coarse fraction of PM_10_. However, Pun and colleagues examined the effects of the chemical constituents of PM_10_ on hospital admissions in Hong Kong and found that NO_3_^−^, K^+^, Cl^−^ and Na^+^ were associated with cardiovascular admissions and SO_4_^2−^, Na^+^ and Mg^2+^ with respiratory admissions [34]. It is noteworthy that the estimated risks to the constituents of PM_2.5–10_ in our study, with the exception of NO_3_^−^, were of similar magnitude to those obtained in Hong Kong, although the concentration of pollutants was quite different. In addition, we observed statistically significant negative associations for Cl^−^ and Na^+^ for both causes of hospitalization. These constituents in the coarse fraction were strongly correlated (0.78) and should be better examined, as they are predominant in this fraction.

Currently, PM_2.5_ is the indicator of greater interest for the assessment of health effects of particulate matter. Effects of this fraction are usually stronger because they can penetrate deeper into the airways of the respiratory tract and reach the alveoli. Many studies have shown that they are associated with morbidity and mortality by cardiovascular and respiratory diseases [1,28,35,36]. In our study, fine particles were only associated with cardiovascular admissions. In a study conducted in four cities of Connecticut and Massachusetts (U.S.), there was a positive association between PM_2.5_ and both causes of hospital admissions, but only the association with cardiovascular diseases was statistically significant [10]. In another study conducted in six cities in the U.S., Lepeule and colleagues found that for every 10 μg/m^3^ of PM_2.5_ the risk of death from cardiovascular diseases increased by 26% (95% CI: 14; 40), an effect of similar magnitude to that observed in our study [2].

Among the constituents of PM_2.5_, K^+^ was positively associated with both outcomes while SO_4_^2−^ and NH_4_^+^ were associated only with circulatory diseases. In Seoul, Korea, Son and colleagues examined the effects of the same constituents and observed a moderate association (*p* < 0.10) for NO_3_^−^ and SO_4_^2−^ and a strong association (*p* < 0.05) for NH_4_^+^ with mortality for cardiovascular diseases and between Cl^−^ and Mg^2+^ and mortality for respiratory disease (*p* < 0.10) [8]. Yet, Cao and colleagues investigated the associations between PM_2.5_ constituents and cardiopulmonary mortality in Xi’an, China, and found that all ions were associated with mortality except Na^+^ e Ca^2+^. However, following adjustment for PM_2.5_ mass, only carbon species, metals, NH_4_^+^, NO_3_^−^ and Cl^−^ remained associated with the outcomes [11]. On the other hand, there are other studies that did not observe any association between NO_3_^−^, SO_4_^2−^ and NH_4_^+^ of PM_2.5_ with cases of morbidity or mortality [37,38]. Bell and colleagues analyzed the associations between components of PM_2.5_ and hospital admissions of elderly individuals for respiratory and cardiovascular diseases. Although NH_4_^+^, NO_3_^−^, SO_4_^2−^, elemental and organic carbon were the main constituents of the total mass of PM_2.5_, only V, Ni and elemental carbon exhibited statistically significant associations with the investigated outcomes, while the soluble ions did not [39].

Emissions of NO_3_^−^, SO_4_^2−^, NH_4_^+^ and K^+^ are mainly due to human activities. The former are secondary species, formed in the atmosphere from precursor gases emitted during the burning of fossil fuels and the latter is emitted from biomass burning [40,41]. It is noteworthy that studies have shown associations between cases of morbidity and mortality from cardiovascular diseases with vehicle emissions [36,42,43], which together with industrial (petrochemical) emissions, are the main source of air pollution in São José dos Campos.

There is great variability in the literature regarding the associations and effect estimates of particles on health. According to Son and colleagues, such variability is probably explained by variations in the chemical composition of the particles that are different from place to place [8]. In turn, Akhtar and colleagues considered that the biological response might result from the combination of the various characteristics of particles, such as mass, size, surface area and chemical composition, for which reason both the physical and chemical properties ought to be taken into account in the explanation of the toxicity of PM_10_ [44]. Moreover, on many occasions, studies employ different methodological criteria in the evaluation of the effects of particles on health, making a comparison of the findings difficult.

An important limitation of the present study is the restricted time frame and chemical constituents available for analysis. A larger period and the evaluation of other chemical constituents of PM_10_ may provide further elements for a better assessment of the effects of particulate matter on the health of this population. In addition, the levels of some chemical constituents could not be assessed on many days due to a very low concentration. This made it impossible to analyze the effects of all of the soluble ions because low concentration levels can be subject to measurement errors and thus interfere with the risk estimates. On the other hand, for the first time, we were able to estimate the hospitalization risks associated with exposure to PM_2.5_ e PM_2.5–10_ and their soluble ions in a vulnerable population group in Latin America. Our results indicate that the risks for different health outcomes vary in relation to the fractions and chemical composition of PM_10_. It is worth mentioning that PM_10_ speciation studies may contribute to the establishment of more selective pollution control policies, i.e., policies aimed at controlling specific emission sources of compounds for which the relationship with morbidity is more evident. Nevertheless, these require a systematic collection of various fractions of PM_10_, as well as a characterization of the chemical composition for the inorganic and organic airborne particulate species.

## 5. Conclusions

The results agree with evidence indicating that the risks for different health outcomes vary in relation to the fractions and chemical composition of PM_10_. Thus, PM_10_ speciation studies may contribute to the establishment of more selective pollution control policies.

## Figures and Tables

**Table 1:** Descriptive statistics of pollutants, weather variables and hospital admissions, São José dos Campos, Brazil, 2010–2011.

Parameter	Mean	SD	Min	P25	P50	P75	Max	T
Inhalable Particulate Matter (μg/m^3^)								
PM_2.5_	4.4	2.4	0.2	2.6	3.9	5.7	15.1	
PM_2.5–10_	8.4	5.6	0.1	4.3	7.1	11.2	29.7	
PM_10_	12.7	7.2	0.7	7.4	11.2	16.7	36.8	
PM_10_^a^	24.5	13.6	6.2	15.2	21.0	30.3	93.6	
Weather variables								
Minimum temperature (°C)	17.5	3.5	8.3	15.1	17.9	20.8	23.7	
Average temperature (°C)	21.9	3.3	13.0	19.3	22.3	24.4	29.0	
Maximum temperature (°C)	28.6	4.6	17.1	25.3	29.0	32.5	37.3	
Minimum humidity (%)	56.9	16.4	20.1	45.9	55.9	66.0	99.1	
Average humidity (%)	81.1	10.0	49.1	75.3	81.9	87.1	99.2	
Hospital admissions (cases/day)								
Circulatory diseases	5	2	0	3	5	7	12	1765
Respiratory diseases	3	2	0	1	3	4	9	972

SD, standard deviation; Min, minimum value; P, percentiles; Max, maximum value; T, total hospital admissions; ^a^ the Environmental Company of the State of São Paulo (Companhia Ambiental do Estado de São Paulo—CETESB) station.

**Table 2:** Descriptive statistics of the PM_2.5_ and PM_2.5–10_ chemical constituents (ng/m^3^). São José dos Campos. Brazil, 2010–2011.

Parameter	Mean	SD	Min	P25	P50	P75	Max
PM_2.5_							
Cl^−^	38.2	24.0	0.7	21.8	38.8	43.1	188.0
NO_3_^-^	79.6	83.4	3.7	35.2	46.5	93.5	599.0
SO_4_^2^^−^	399.2	314.4	0.6	186.4	320.3	530.3	1957.5
Na^+^	40.4	39.4	1.4	20.6	25.1	44.6	285.6
NH_4_^+^	98.3	94.8	0.3	43.7	63.4	120.7	636.8
K^+^	26.7	24.9	0.1	9.0	23.0	31.4	208.6
Ca^2+^	50.9	55.5	0.1	25.0	36.3	47.4	542.3
Mg^2+^	14.2	14.1	0.1	3.5	8.3	28.3	88.4
PM_2.5–10_							
Cl^−^	82.7	119.3	2.1	31.3	44.2	90.8	1074.0
NO_3_^-^	284.7	295.0	5.2	106.4	202.7	384.3	3566.5
SO_4_^2^^−^	158.7	149.9	0.2	64.4	110.5	212.9	1286.4
Na^+^	79.2	101.2	1.2	25.8	50.7	96.6	1006.5
NH_4_^+^	45.3	65.1	0.8	20.2	37.5	50.6	896.1
K^+^	31.5	36.3	0.6	14.3	21.2	32.4	282.8
Ca^2+^	167.9	176.4	7.6	62.2	121.4	216.9	2157.8
Mg^2+^	28.5	23.6	0.2	13.0	24.4	38.4	307.4

SD, standard deviation; Min, minimum value; P, percentiles; Max, maximum value.

**Table 3:** Relative Risk percentage (%RR) and confidence interval (95% CI) in polynomial distributed lag model (PDLM) (overall effect within 0–5 days) to elderly hospital admissions for respiratory and circulatory causes.

Pollutants	Respiratory Diseases %RR (LL, UL)	Circulatory Diseases %RR (LL, UL)
PM_2.5_	8.5 (−6.8, 26.3)	19.6 (6.4, 34.6)
PM_2.5–10_	23.5 (13.5, 34.3)	0.8 (−5.8, 7.7)
PM_10_	12.8 (6.0, 20.0)	2.7 (−2.2, 7.9)
PM_10_^a^	8.9 (5.2, 12.8)	1.2 (−1.7, 4.0)
Soluble ions (PM_2.5_)		
SO_4_^2−^	0.0 (−0.1, 0.1)	0.2 (0.1, 0.3)
NH_4_^+^	−0.3 (−0.7, 0.1)	1.2 (1.0, 1.5)
K^+^	2.7 (1.1, 4.3)	1.6 (0.3, 2.8)
Soluble ions (PM_2.5–10_)		
Cl^−^	−0.5 (−0.8, −0.2)	−0.7 (−0.9, −0.4)
NO_3_^-^	−0.1 (−0.2, 0.1)	0.2 (0.1, 0.3)
SO_4_^2−^	0.4 (0.1, 0.6)	0.8 (0.6, 1.0)
Na^+^	−0.4 (−0.8, 0.0)	−0.3 (−0.6, 0.0)
K^+^	−0.2 (-1.2, 0.8)	1.0 (0.2, 1.8)
Ca^2+^	0.0 (−0.2, 0.2)	0.1 (−0.1, 0.3)
Mg^2+^	0.1 (−1.5, 1.8)	0.4 (−0.9, 1.7)

LL, lower limit; UL, upper limit; ^a^ Cetesb station; in bold *p*-value < 0.05.

## References

[1] Dominici F., Peng R.D., Bell M.L., Pham L., McDermott A., Zeger S.L., Samet J.M. (2006). Fine particulate air pollution and hospital admission for cardiovascular and respiratory diseases. JAMA.

[2] Lepeule J., Laden F., Dockery D., Schwartz J. (2012). Chronic exposure to fine particles and mortality: An extended follow-up of the Harvard Six Cities Study from 1974 to 2009. Environ. Health Perspect..

[3] Ostro B., Roth L., Malig B., Marty M. (2009). The effects of fine particle components on respiratory hospital admissions in children. Environ. Health Perspect..

[4] Gomes M.J.M. (2002). Ambiente e pulmão. J. Pneumol..

[5] Halonen J.I., Lanki T., Yli-Tuomi T., Tiittanen P., Kulmala M., Pekkanen J. (2009). Particulate air pollution and acute cardiorespiratory hospital admissions and mortality among the elderly. Epidemiology.

[6] Meister K., Johansson C., Forsberg B. (2012). Estimated short-term effects of coarse particles on daily mortality in Stockholm, Sweden. Environ. Health Perspect..

[7] Bell M.L., Dominici F., Ebisu K., Zeger S.L., Samet J.M. (2007). Spatial and temporal variation in PM_2.5_ chemical composition in the United Statess for health effects studies. Environ. Health Perspect..

[8] Son J.Y., Lee J.T., Kim K.H., Jung K., Bell M.L. (2012). Characterization of fine particulate matter and associations between particulate chemical constituents and mortality in Seoul, Korea. Environ. Health Perspect..

[9] Zanobetti A., Franklin M., Koutrakis P., Schwartz J. (2009). Fine particulate air pollution and its components in association with cause-specific emergency admissions. Environ. Health..

[10] Bell M.L., Ebisu K., Leaderer B.P., Gent J.F., Lee H.J., Koutrakis P., Wang Y., Dominici F., Peng R.D. (2014). Associations of PM_2.5_ constituents and sources with hospital admissions: Analysis of four counties in Connecticut and Massachusetts (USA) for persons ≥65 years of age. Environ. Health Perspect..

[11] Cao J., Xu H., Xu Q., Chen B., Kan H. (2012). Fine particulate matter constituents and cardiopulmonary mortality in a heavily polluted Chinese city. Environ. Health Perspect..

[12] McCracken J., Baccarelli A., Hoxha M., Dioni L., Melly S., Coull B., Suh H., Vokonas P., Schwartz J. (2010). Annual ambient black carbon associated with shorter telomeres in elderly men: Veterans affairs normative aging study. Environ. Health Perspect..

[13] Cassee F.R., Héroux M.E., Gerlofs-Nijland M.E., Kelly F.J. (2013). Particulate matter beyond mass: Recent health evidence on the role of fractions, chemical constituents and sources of emission. Inhal. Toxicol..

[14] Romieu I., Gouveia N., Cifuentes L.A., Leon A.P., Junger W., Vera J., Strappa V., Hurtado-Díaz M., Miranda-Soberanis V., Rojas-Bracho L. (2012). Multicity study of air pollution and mortality in Latin America (the ESCALA Study). Res. Rep. Health Eff. Inst..

[15] O Vale S. José Faz Cadastramento Para Restringir “Forasteiros” na Saúde. http://www.ovale.com.br/nossa-regi-o/s-jose-faz-cadastramento-para-restringir-forasteiros-na-saude-1.223717.

[16] DATASUS Departamento de Informática do SUS. http://www2.datasus.gov.br/DATASUS/index.php.

[17] Hopke P.K., Xie Y., Raunemma T., Biegalski S., Landsberger S., Maenhaut W., Artaxo P., Cohen D. (1997). Characterization of the Gent stacked filter unit PM_10_ sampler. Aerosol Sci. Technol..

[18] Ferreira T.M., Forti M.C., Alvalá P.C. Protocolo Para Coleta de Material Particulado Atmosférico. http://urlib.net/8JMKD3MGP7W/3B9PGQL.

[19] Forti M.C., Alcaide R.L.M. Validação de Métodos Analíticos do Laboratório de Aerossóis, Soluções Aquosas e Tecnologias—LAQUATEC. http://urlib.net/8JMKD3MGP7W/39QJ7P2.

[20] Forti M.C., Alcaide R.L.M. Protocolo de Determinação de ânions Inorgânicos em Soluções Aquosas Por Cromatografia iônica. http://urlib.net/8JMKD3MGP7W/3B86KQ5.

[21] Forti M.C., Alcaide R.L.M. Protocolo de Determinação de Cátions Inorgânicos em Soluções Aquosas Por Cromatografia Liquída de íons. http://urlib.net/8JMKD3MGP7W/3FJFPGL.

[22] Junger W.L., Ponce de Leon A. (2015). Imputation of missing data in time series for air pollutants. Atmos. Environ..

[23] Schwartz J., Spix C., Touloumi G., Bachárová L., Barumamdzadeh T., Tertre A.L., Piekarksi T., Ponce de Leon A., Ponka A., Rossi G. (1996). Methodological issues in studies of air pollution and daily counts of deaths or hospital admissions. J. Epidemiol. Community Health..

[24] Zanobetti A., Wand M.P., Schwartz J., Ryan L.M. (2000). Generalized additive distributed lag models: Quantifying mortality displacement. Biostatistics.

[25] Junger W.L. (2008). Análise, Imputação de Dados e Interfaces Computacionais em Estudos de Séries Temporais Epidemiológicas. Doutorado Thesis.

[26] World Health Organization (WHO) (2006). Air Quality Guidelines for Particulate Matter, Ozone, Nitrogen Dioxide and Sulfur Dioxide. Global Update 2005. Summary of Risk Assessment.

[27] Anderson J.O., Thundiyil J.G., Stolbach A. (2012). Clearing the air: A review of the effects of particulate matter air pollution on human health. J. Med. Toxicol..

[28] Beelen R., Raaschou-Nielsen O., Stafoggia M., Andersen Z.J., Weinmayr G., Hoffmann B., Wolf K., Samoli E., Fischer P., Nieuwenhuijsen M. (2014). Effects of long-term exposure to air pollution on natural-cause mortality: An analysis of 22 European cohorts within the multicentre ESCAPE project. Lancet.

[29] Hoek G., Krishnan R.M., Beelen R., Peters A., Ostro B., Brunekreef B., Kaufman J.D. (2013). Long-term air pollution exposure and cardio-respiratory mortality: A review. Environ. Health.

[30] Nardocci A.C., Freitas C.U., Leon A.C.M.P., Junger W.L., Gouveia N.C. (2013). Poluição do ar e doenças respiratórias e cardiovasculares: Estudo de séries temporais em Cubatão, São Paulo, Brasil. Cad. Saude Publica..

[31] Nascimento L.F.C., Francisco J.B., Patto M.B.R., Antunes A.M. (2012). Environmental pollutants and stroke-related hospital admissions. Cad. Saude Publica..

[32] Malig B.J., Green S., Basu R., Broadwin R. (2013). Coarse particles and respiratory emergency department visits in California. Am. J. Epidemiol..

[33] Stafoggia M., Samoli E., Alessandrini E., Cadum E., Ostro B., Berti G., Faustini A., Jacquemin B., Linares C., Pascal M. (2013). Short-term associations between fine and coarse particulate matter and hospitalizations in Southern Europe: Results from the MED-PARTICLES Project. Environ. Health Perspect..

[34] Pun V.C., Yu I.T., Qiu H., Ho K.-F., Sun Z., Louie P.K.K., Wong T.W., Tian L. (2014). Short-term associations of cause-specific emergency hospitalizations and particulate matter chemical components in Hong Kong. Am. J. Epidemiol..

[35] Brook R.D., Rajagopalan S., Pope C.A., Brook J.R., Bhatnagar A., Diez-Roux A.V., Holguin F., Hong Y., Luepker R.V., Mittlemanet M.A. (2010). Statement from the American Heart Association particulate matter air pollution and cardiovascular disease: An update to the scientific. Circulation.

[36] Ito K., Mathes R., Ross Z., Nádas A., Thurston G., Matte T. (2011). Fine particulate matter constituents associated with cardiovascular hospitalizations and mortality in New York City. Environ. Health Perspect..

[37] Kim S.-Y., Peel J.L., Hannigan M.P., Dutton S.J., Sheppard L., Clark M.L., Veda S. (2012). The temporal lag structure of short-term associations of fine particulate matter chemical constituents and cardiovascular and respiratory hospitalizations. Environ. Health Perspect..

[38] Krall J.R., Anderson G.B., Dominici F., Bell M.L., Peng R.D. (2013). Short-term exposure to particulate matter constituents and mortality in a national study of U.S. urban communities. Environ. Health Perspect..

[39] Bell M.L., Ebisu K., Peng R.D., Samet J.M., Dominici F. (2009). Hospital admissions and chemical composition of fine particle air pollution. Am. J. Respir. Crit. Care Med..

[40] Andrade M.F., Miranda R.M., Fornaro A., Kerr A., Oyama B., Andre P.A., Saldiva P. (2010). Vehicle emissions and PM_2.5_ mass concentrations in six Brazilian cities. Air Qual. Atmos. Health..

[41] Souza P.A., Mello W.Z., Mariani R.L., Sella S.M. (2010). Caracterização do material particulado fino e grosso e composição da fração Inorgânica solúvel em água em São José dos Campos (SP). Quim. Nova..

[42] Gan W.Q., FitzGerald J.M., Carlsten C., Sadatsafavi M., Brauer M. (2013). Associations of ambient air pollution with chronic obstructive pulmonary disease hospitalization and mortality. Am. J. Respir. Crit. Care Med..

[43] Pun V.C., Yu I.T., Ho K.-F., Qiu H., Sun Z., Tian L. (2014). Differential effects of source-specific particulate matter on emergency hospitalizations for ischemic heart disease in Hong Kong. Environ. Health Perspect..

[44] Akhtar U.S., Rastogi N., McWhinney R.D., Urch B., Chow C.-W., Evans G.J., Scott J.A. (2014). The combined effects of physicochemical properties of size-fractionated ambient particulate matter on in vitro toxicity in human A549 lung epithelial cells. Toxicol. Rep..

